# Ischemic Postconditioning Protects Against Intestinal Ischemia/Reperfusion Injury via the HIF-1α/miR-21 Axis

**DOI:** 10.1038/s41598-017-16366-6

**Published:** 2017-11-23

**Authors:** Zhongzhi Jia, Weishuai Lian, Haifeng Shi, Chuanwu Cao, Shilong Han, Kai Wang, Maoquan Li, Xiaoping Zhang

**Affiliations:** 1 Department of Interventional Radiology, No. 2 People’s Hospital of Changzhou, Nanjing Medical University, Chang zhou, 213003 China; 2 Department of Interventional Radiology, Shanghai Tenth People’s Hospital, Shanghai, 200072 China; 3 Institute of Medical Intervention Engineering, Tongji University, Shanghai, 200072 China

## Abstract

Intestinal ischemia/reperfusion (I/R) can lead to tissue damage associated with inflammation and mucosal apoptosis. Ischemic postconditioning (IPostC), a series of repeated, brief, intermittent periods of ischemia and reperfusion, has beneficial effects against I/R-induced injury in the heart and intestine, although the underlying mechanisms for these effects remain unclear. We evaluated the involvement of microRNA-21 (miR-21) in the protective effects of IPostC in a rat model of I/R induced by superior mesenteric artery occlusion and reopening. IPostC decreased I/R injury and suppressed apoptosis in the intestinal tissues concomitant with the induction of hypoxia inducible factor 1 alpha (HIF-1α) and the upregulation of miR-21. *In vitro* experiments in the intestinal epithelial cell line IEC-6 showed that hypoxia induced miR-21 and this effect was abolished by silencing HIF1-α, confirming the induction of miR-21 by HIF1-α, HIF1-α or miR-21 inhibition exacerbated I/R induced apoptosis, and programmed cell death 4 (PDCD4) and Fas-L was involved in miR-21 mediated anti-apoptotic effects on intestinal epithelial cells. Knockdown of miR-21 or inhibition of HIF1-α abolished the IPostC-mediated attenuation of intestinal injury and apoptosis and the downregulation of PDCD4 and Fas-L. A potential mechanism underlying the protective effect of IPostC may therefore involve the induction of miR-21 by HIF1-α and the attenuation of apoptosis via the downregulation of PDCD4 and Fas-L.

## Introduction

Intestinal ischemia/reperfusion (I/R) is a serious condition associated with several disorders such as mesenteric ischemia, infection, and shock; this condition is also associated with surgical procedures^1^. Reperfusion of ischemic tissues activates innate immune responses and leads to inflammation, which in turn leads to hypoxic cell damage^2^. Intestinal I/R injury results in damage to the intestinal mucosa, impairment of the local microvasculature, increased vascular and mucosal permeability, and multiple organ failure, which is associated with a high mortality rate (67–80%)^3,4^.

Ischemic postconditioning (IPostC), which consists of repeated brief intermittent periods of ischemia and reperfusion, is applied in the early phase of reperfusion after prolonged ischemia^5^. IPostC has been shown to attenuate I/R injury in the heart by limiting infarct size, decreasing apoptosis, improving vascular endothelial dysfunction, and preventing heart failure^6^. In I/R-induced intestinal injury, IPostC inhibits oxidative damage and neutrophil infiltration and attenuates inflammation^7–9^. However, the exact mechanism by which IPostC confers intestinal protection from intestinal I/R injury remains to be elucidated.

Several microRNAs (miRNAs) have been implicated in the regulation of I/R injury^6^. miR-21 is upregulated by IPostC and has a protective effect against I/R injury in the heart through target programmed cell death 4 (PDCD4)^10^. The role of miRNAs in IPostC was further demonstrated in a study by He *et al*.^11^, which showed the upregulation of miR-1 and miR-133 by IPostC during reperfusion in a rat model. miR-21 has also been shown to be upregulated by IPostC in the myocardium, alleviating I/R-induced cardiomyocyte apoptosis through modulation of the PTEN/Akt signaling pathway^6^. Additionally, IPostC-mediated regulation of miR-1 and miR-21 have been found to attenuate apoptosis in patients undergoing valvular heart surgery^12^.

Hypoxia inducible factor 1 (HIF-1) is a transcription factor that is expressed under conditions of hypoxia and activates genes related to angiogenesis, glycolysis, vascular tone regulation, and erythropoiesis^13^. HIF-1α upregulation occurs in certain organs after exposure to hypoxia and is an early response to myocardial ischemia in humans, leading to angiogenesis and improving myocardial cell survival^14^. The expression of HIF-1α was significantly increased in the ischemic intestinal mucosa^15^. We also found that the expression of HIF-1α increased further after Ischemic postconditioning. However, little is known as to how the role of HIF-1α and miR-21 in IPostC protects against intestinal ischemia/reperfusion injury.

In the present study, we examined the involvement of HIF-1α and miR-21 in the progression of intestinal I/R in a rat model and explored the molecular mechanisms underlying the protective effects of IPostC after intestinal I/R.

## Results

### IPostC attenuated intestinal I/R injury and was associated with the upregulation of HIF-1α and miR-21

**Figure 1:**
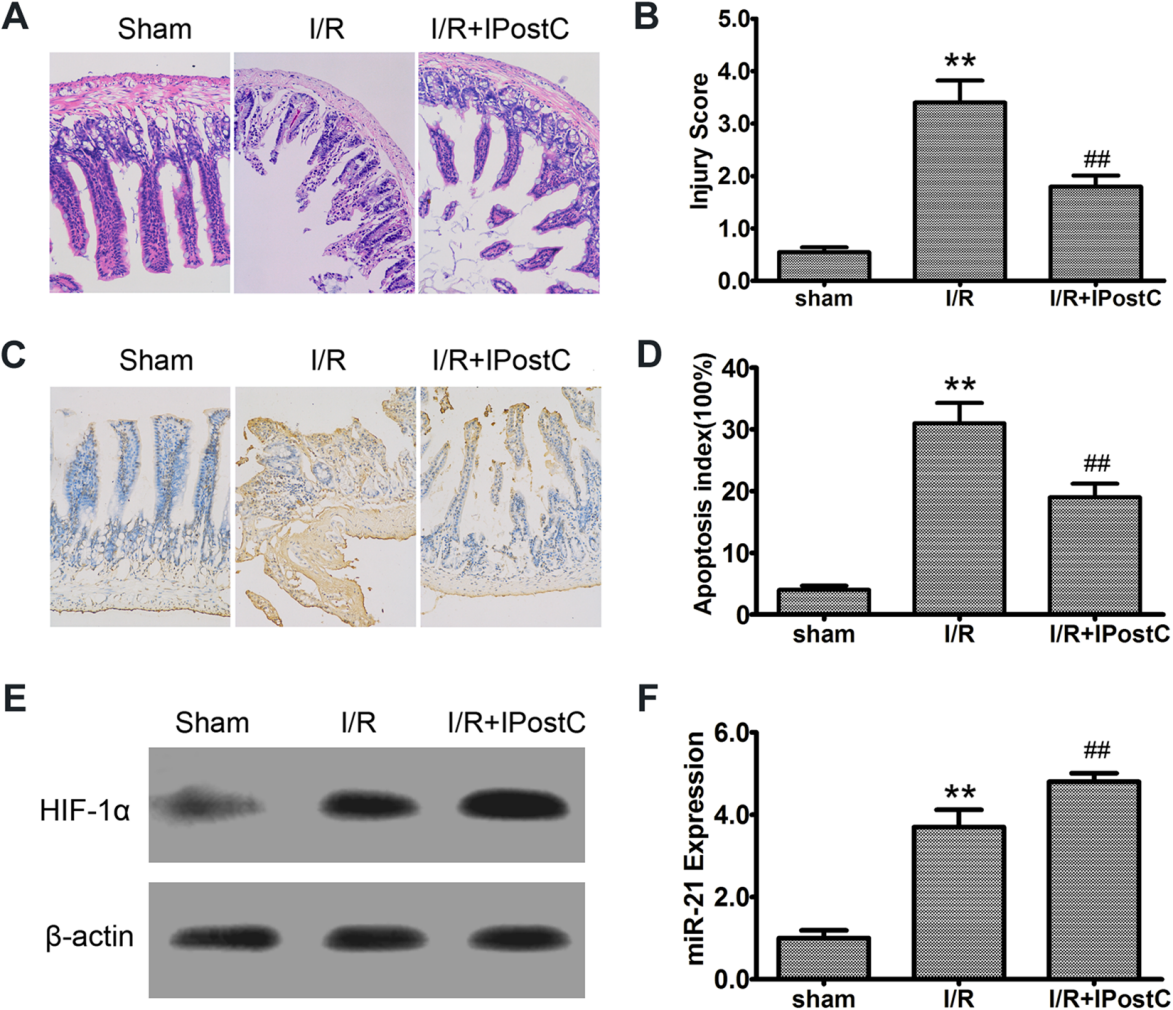
IPostC attenuated intestinal I/R injury and affected expression of HIF-1α and miR-21. (**A**) Hematoxylin and eosin (HE) staining was performed to analyze intestinal injury. Magnification × 200. (**B**) HE-stained, formalin-fixed tissue sections were scored for intestinal epithelial damage (0–6). (**C** and **D**) Evaluation of apoptosis by TUNEL staining and measurement of the apoptotic index. Magnification × 200. (**E**) Western blot analysis of HIF-1α expression in intestinal tissues. (**F**) qRT-PCR analysis of miR-21 expression in the indicated groups. U6 snRNA served as an internal control. Unprocessed original scans and of blots are shown in Supplementary Fig. S1. Data are expressed as mean ± SD, n = 6. ^**^ *P* < 0.01 compared with sham group; ^##^ *P* < 0.01 compared with I/R group.

To investigate the effect of IPostC in I/R injury, we compared intestinal mucosal injuries sustained in the I/R and I/R + IPostC groups. Figure 1A shows representative photomicrographs of HE-stained intestinal tissues from sham-operated rats and from those subjected to I/R and I/R + IPostC. I/R significantly increased intestinal mucosal injury scores compared with the sham operation (3.67 ± 0.37 vs 0.42 ± 0.08; *P* < 0.01), and IPostC decreased the injury score in rats after I/R versus I/R alone (1.86 ± 0.27; *P* < 0.01) (Fig. 1B). Evaluation of apoptosis by TUNEL staining demonstrated few TUNEL-positive (apoptotic) cells in the sham group, with an increase in TUNEL-positive cells in the I/R group and a decrease in the IPostC group (Fig. 1C). Quantification of apoptosis demonstrated an approximately 7.5-fold increase in the apoptotic index in the I/R group compared with the index in the sham group (31.3 ± 3.3% vs 4.3 ± 0.7%; *P* < 0.01), with a decrease in the IPostC group (19.1 ± 2.2% vs 31.3 ± 3.3%; *P* < 0.01 vs. I/R) (Fig. 1D). Western blot and qRT-PCR analyses demonstrated that HIF-1α and miR-21 expression in intestinal tissues was upregulated by I/R and further upregulated by IpostC (3.59 ± 0.40, 4.81 ± 0.29 vs 1.0 ± 0.16; *P* < 0.01) (Fig. 1E and F).

### Hypoxia induced HIF-1α and miR-21 protected against apoptosis in intestinal epithelial cells

**Figure 2:**
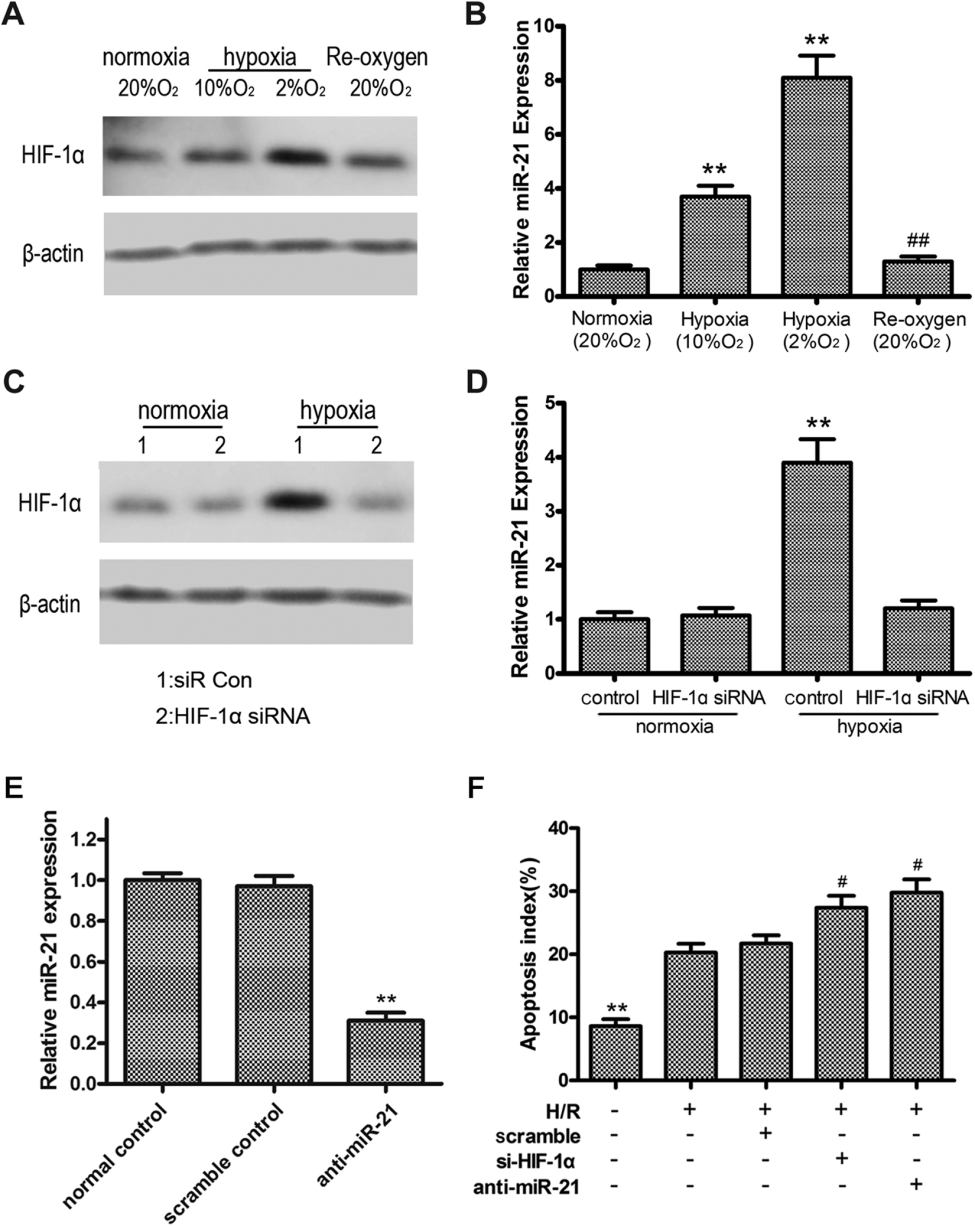
HIF-1α and miR-21 were induced by hypoxia and play an anti-apoptosis role in intestinal epithelial cells. (**A**) Western blot analysis of HIF-1α expression in response to normoxia, hypoxia, and reoxygenation. (**B**) qRT-PCR analysis of miR-21 expression using U6 snRNA as an internal control. (**C**) Western blot assessment of HIF-1α expression in cells transfected with siRNA against HIF-1α or scrambled control. β-actin served as a loading control. Unprocessed original scans and of blots are shown in Supplementary Fig. S1. (**D**) qRT-PCR analysis of miR-21 expression. U6 snRNA served as an internal control. ^**^ *P* < 0.01 compared with normoxia; ^##^ *P* < 0.01 compared with hypoxia (2% O_2_). (**E**) qRT-PCR analysis of miR-21 expression in cells transfected with anti-miR-21 or scrambled control under H/R treatment. (**F**) Effect of HIF-1α and miR-21 on H/R induced apoptosis in intestinal epithelial cells. ^**^ *P* < 0.01 and ^#^ *P* < 0.05 vs H/R group. Each value represents the average of 3 independent experiments, and error bars represent the SD of the mean in triplicate.

The roles of HIF-1α and miR-21 in response to hypoxia were examined in the intestinal epithelial cell line IEC-6 by assessing HIF-1α and miR-21 expression under normoxic (20% O_2_) and hypoxic (10% or 2% O_2_) conditions and during hypoxia/reoxygenation (H/R). As shown in Fig. 2A, low oxygen induced HIF-1α expression, whereas re-oxygenation restored HIF-1α levels, confirming HIF-1α as a positive indicator of hypoxia. The effect of hypoxia on miR-21 expression in IEC-6 cells was analyzed with qRT-PCR. In parallel with HIF-1α expression, miR-21 was significantly upregulated by hypoxia and its levels were restored by re-oxygenation (Relative 3.7 ± 0.41, 8.1 ± 0.79, 1.3 ± 0.17 vs sham) (Fig. 2B). siRNA-mediated silencing of HIF-1α in IE-6 cells abolished the effect of hypoxia on the upregulation of HIF-1α and miR-21 expression, as determined by western blotting and qRT-PCR (Relative 1.2 ± 0.15 vs. 3.9 ± 0.43; *P* < 0.01) (Fig. 2C and D), suggesting that the upregulation of miR-21 by hypoxia is mediated by HIF-1α.

To determine the role of HIF-1α and miR-21 in intestinal I/R injury, a cell H/R model was applied. Intestinal epithelial cell line IEC-6 was transfected with si-HIF-1α, anti-miR-21 or scrambled control. We found that miR-21 expression was successfully inhibited by anti-miR-21 (0.31 ± 0.03 vs 1.0 ± 0.12; *P* < 0.01), in cultured intestinal epithelial cells (Fig. 2E). As expected, H/R resulted in a 1.4- fold increase in apoptosis (20.3 ± 1.5 vs 8.6 ± 1.1, *P* < 0.01; Fig. 2F). Notably, cell apoptosis was exacerbated after treatment with si-HIF-1α or anti-miR-21 (27.4 ± 1.9 vs 8.6 ± 1.1, 29.8 ± 2.1 vs 8.6 ± 1.1, respectively, *P* < 0.05; Fig. 2F). In contrast, control oligo (scramble) had no effect on H/R-mediated cell apoptosis. These results indicated that IPostC-upregulated HIF-1α/miR-21 had a protective effect against ischaemia-induced intestinal epithelial cell apoptosis *in vitro*.

### PDCD4 and Fas-L was involved in miR-21 mediated anti-apoptotic effects on intestinal epithelial cells

**Figure 3:**
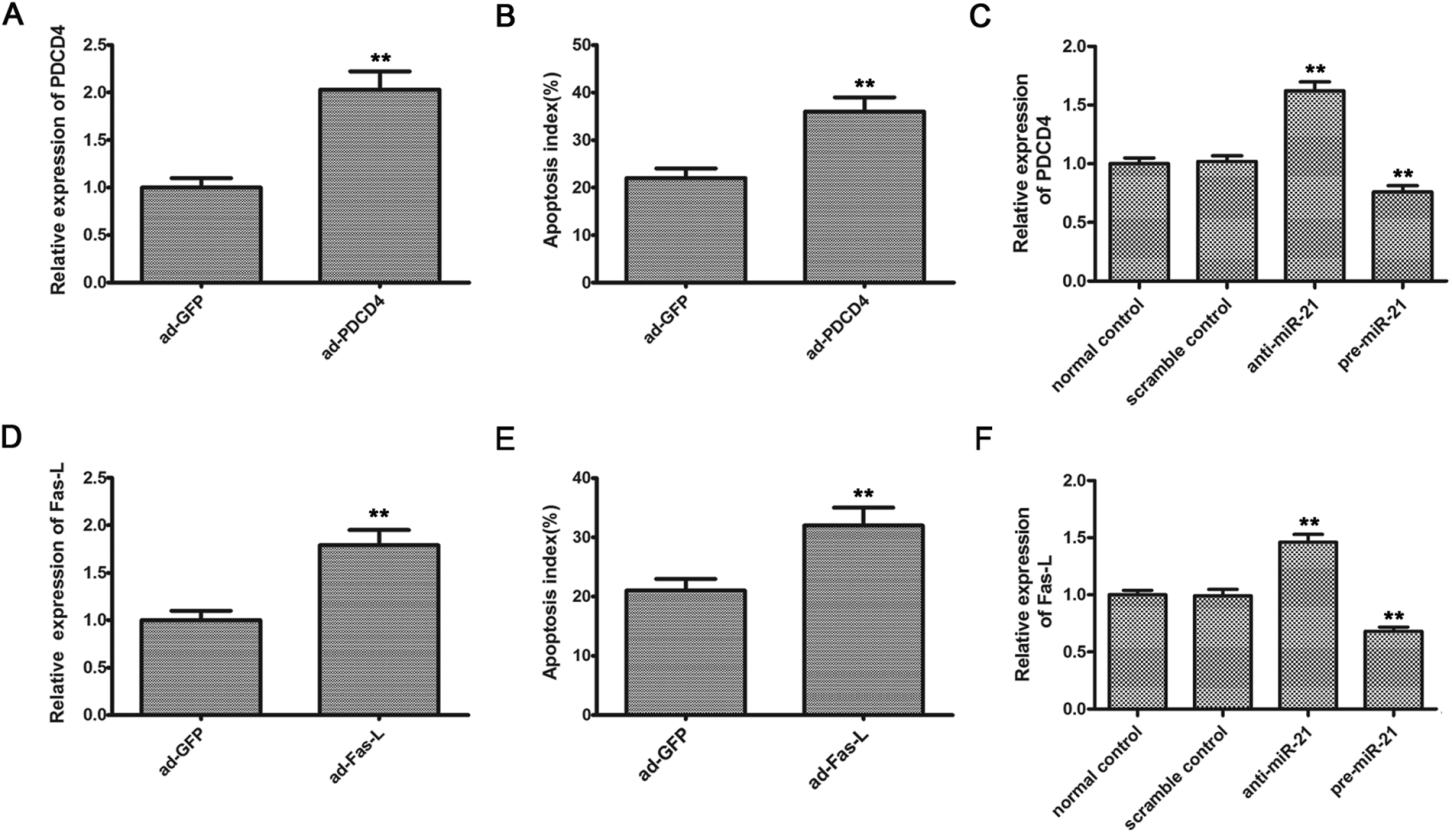
PDCD4 and Fas-L was involved in miR-21 mediated anti-apoptotic effects on intestinal epithelial cells. (**A**) Overexpression of PDCD4 by Ad-PDCD4 (30 MOI) in intestinal epithelial cells. (**B**) Ad-PDCD4 (30 MOI) increased intestinal epithelial cell apoptosis induced by H/R as determined by TUNEL staining. (**C**) Modulation of PDCD4 expression in intestinal epithelial cells by anti-miR-21 (30 nM) and pre-miR-21 (30 nM). (**D**) Overexpression of Fas-L by Ad-Fas-L (30 MOI) in intestinal epithelial cells. (**E**) Ad-Fas-L (30 MOI) increased intestinal epithelial cell apoptosis induced by H/R as determined by TUNEL staining. (**F**) Modulation of Fas-L expression in intestinal epithelial cells by anti-miR-21 (30 nM) and pre-miR-21 (30 nM). Data are expressed as mean ± SD. n = 3. ^**^ *P* < 0.01 compared with Ad-GFP control in A, B, D and E. ^*^ *P* < 0.05 and ^**^ *P* < 0.01 compared with normal control in (**C** and **F**).

PDCD4 and Fas-L are potential target genes of miR-21 as described in previous studies^10,16^. The role of PDCD4 and Fas-L in intestinal epithelial cell apoptosis is currently unclear. To test it, we overexpressed PDCD4 and Fas-L by Ad-PDCD4 and Ad-Fas-L in cultured intestinal epithelial cells. As shown in Fig. 3A, PDCD4 expression was increased by Ad-PDCD4 in these cells. Indeed, consistent with PDCD4 expression, Ad-PDCD4 increased intestinal epithelial cell apoptosis induced by I/R (36.0 ± 3.1% vs 22.1 ± 2.3%; *P* < 0.01) (Fig. 3B). In addition, PDCD4 expression in intestinal epithelial cells was upregulated by anti-miR-21 (1.62 ± 0.07 vs 1.0 ± 0.05, *P* < 0.01), but downregulated by pre-miR-21 (0.76 ± 0.04 vs 1.0 ± 0.05, *P* < 0.01) (Fig. 3C). As shown in Fig. 3D, Fas-L expression was increased by Ad-Fas-L in intestinal epithelial cells, and consistent with Fas-L expression, Ad-Fas-L increased intestinal epithelial cell apoptosis induced by I/R (32.1 ± 2.0% vs 21.3 ± 2.2%; *P* < 0.01) (Fig. 3E). As well, Fas-L expression in intestinal epithelial cells was upregulated by anti-miR-21 (1.46 ± 0.06 vs 1.0 ± 0.04, *P* < 0.01), but downregulated by pre-miR-21 (0.68 ± 0.03 vs 1.0 ± 0.04, *P* < 0.01) (Fig. 3F). The results suggest that PDCD4 and Fas-L are involved in miR-21 mediated anti-apoptotic effects on intestinal epithelial cells.

### Knockdown of miR-21 abolished the effects of IPostC on alleviating I/R injury and suppressing apoptosis by upregulating PDCD4 and Fas-L

**Figure 4:**
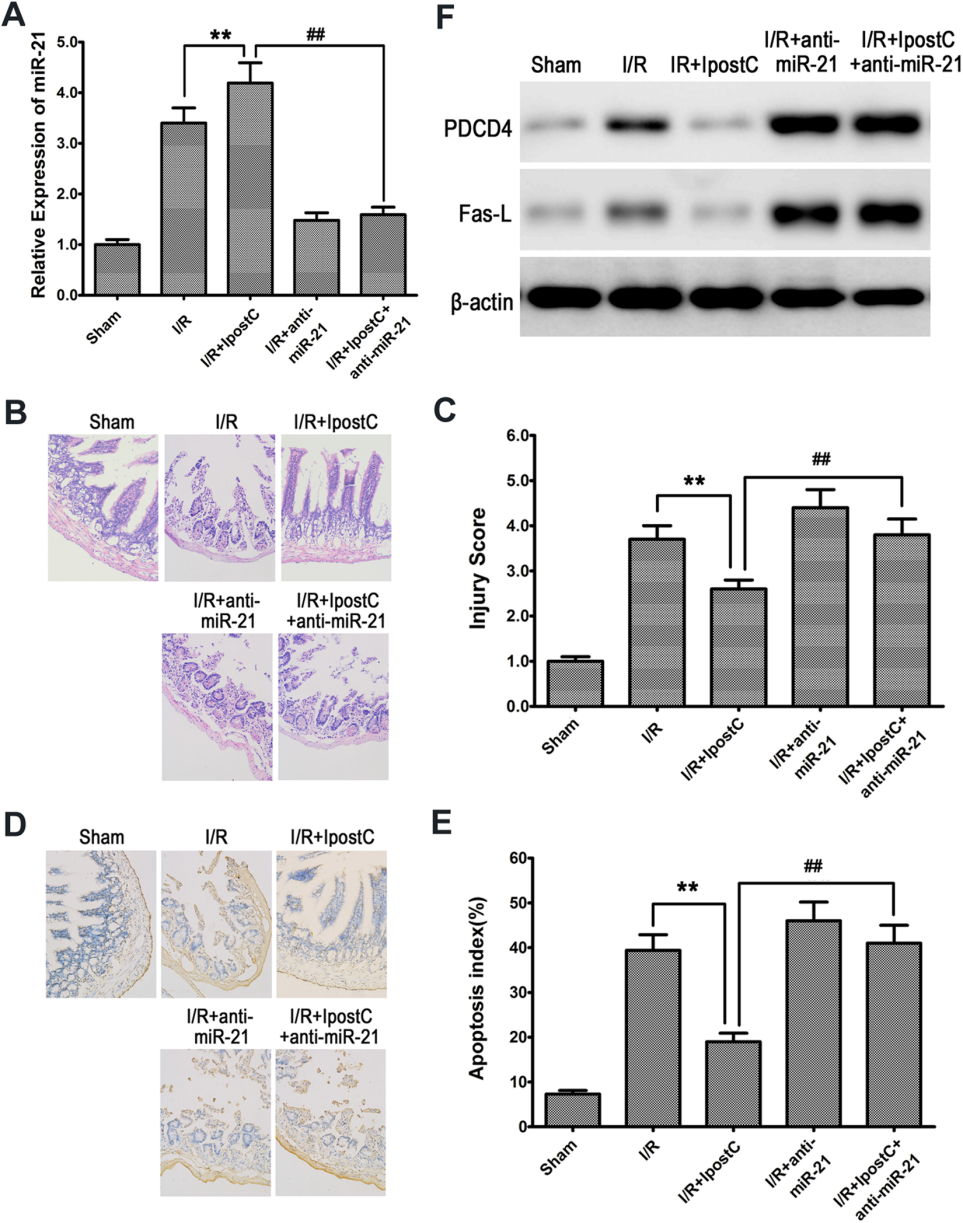
Knockdown of miR-21 abolished the effects of IPostC on alleviating I/R injury and suppressing apoptosis. (**A**) qRT-PCR analysis of miR-21 expression. (**B**) Representative photomicrographs of HE-stained intestinal sections. Magnification ×200. (**C**) Intestinal injury score. (**D** and **E**) Analysis of apoptosis by TUNEL staining and measurement of apoptotic index. Magnification × 200. (**F**) Western blot analysis of PDCD4 and Fas-L expression. β-actin was used as the loading control. Unprocessed original scans and of blots are shown in Supplementary Fig. S1. Data are expressed as mean ± SD, n = 6. ^**^ *P < *0.01 compared with I/R group; ^##^ *P < *0.01 compared with I/R + IPostC group.

To further explore the biological involvement of miR-21 in IPostC-mediated intestinal protection, miR-21 expression was blocked through the delivery of antagomir-21 or scramble antagomir into the intestines of rats 24 hours before IPostC. After 3 hours of reperfusion, miR-21 was upregulated in the intestinal mucosa in IPostC-pretreated rats relative to the I/R group (relative 4.2 ± 0.39 vs 3.4 ± 0.31; *P* < 0.01), and this effect was abolished by miR-21 knockdown (1.59 ± 0.14 vs 4.2 ± 0.39; *P* < 0.01), as determined by qRT-PCR (Fig. 4A). Determination of the injury score in HE-stained sections showed that IPostC significantly attenuated I/R-induced intestinal injury at 3 hours of reperfusion (2.6 ± 0.20 vs 3.7 ± 0.31; *P* < 0.01), and anti-miR-21 abolished these protective effects (3.8 ± 0.35 vs 2.6 ± 0.20; *P* < 0.01) (Fig. 4B and C). Assessment of apoptosis by TUNEL staining showed a similar pattern in which IPostC attenuated apoptosis (19.0 ± 2.1% vs 39.4 ± 3.5%; *P* < 0.01), whereas knockdown of miR-21 suppressed the protective effects of IPostC, restoring the apoptotic index to that of tissues exposed to I/R (41.0 ± 0.39% vs 19.0 ± 2.1%; *P* < 0.01) (Fig. 4D and E). Western blot analysis showed that PDCD4 and Fas-L were downregulated by IPostC, whereas their levels were restored by miR-21 knockdown (Fig. 4F). Taken together, these results indicated that the protective effects of IPostC on I/R-induced intestinal injury may be mediated by the modulation of apoptosis by miR-21 and its target PDCD4.

### HIF-1α inhibition downregulated miR-21 and abolished the protective effects of IPostC mediated by the inhibition of apoptosis

**Figure 5:**
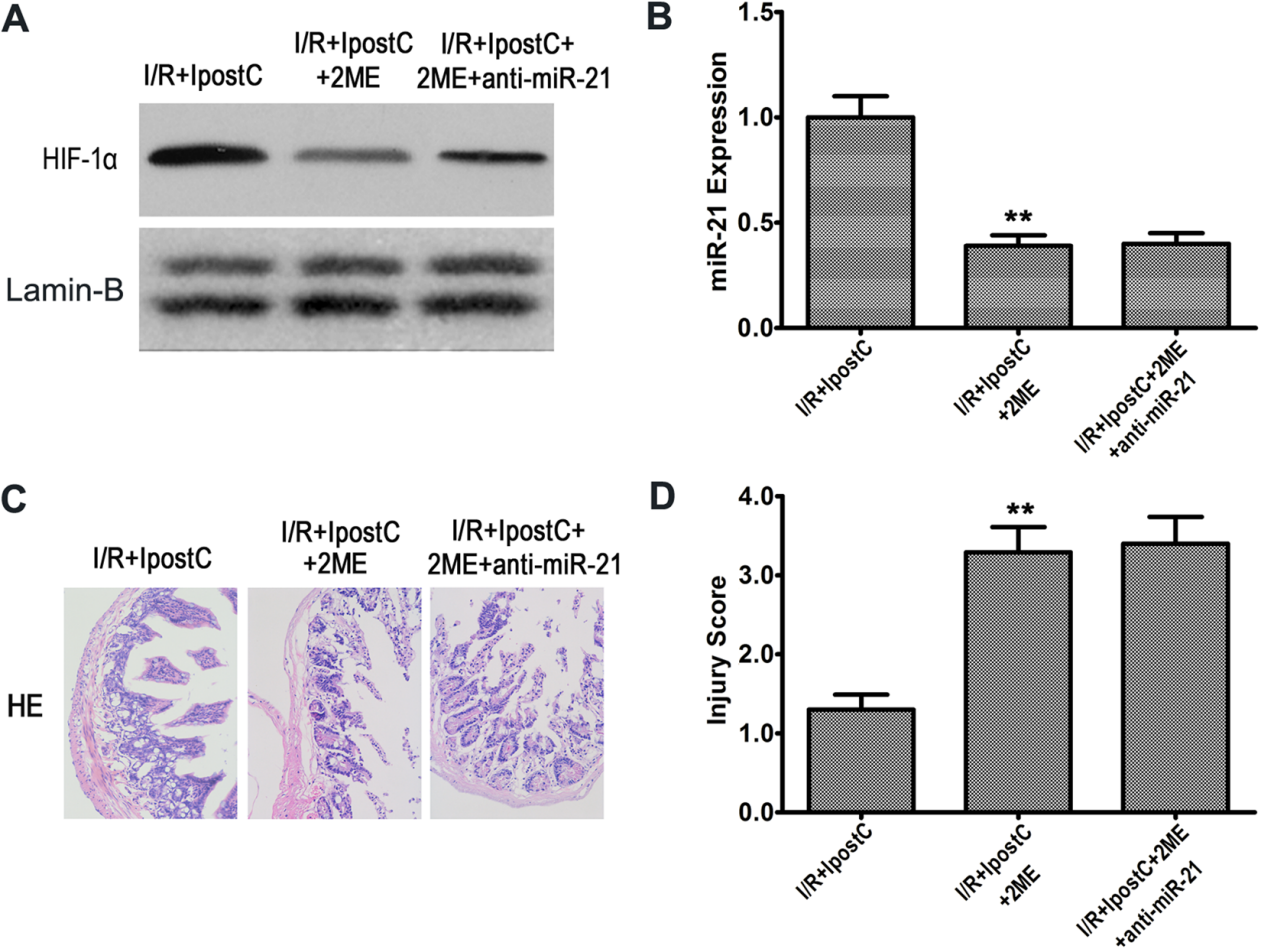
HIF-1α inhibition downregulated miR-21 and abolished the protective effects of IPostC. (**A**) Western blot analysis of HIF-1α expression in the nucleus. (**B**) qRT-PCR analysis of miR-21 expression using U6 snRNA as an internal control. (**C** and **D**) Representative photomicrographs of HE-stained intestinal sections and intestinal injury score. Magnification × 200. Unprocessed original scans and of blots are shown in Supplementary Fig. S1. Data are expressed as mean ± SD, n = 6. ^**^ *P* < 0.01 vs I/R + IPostC group.

**Figure 6:**
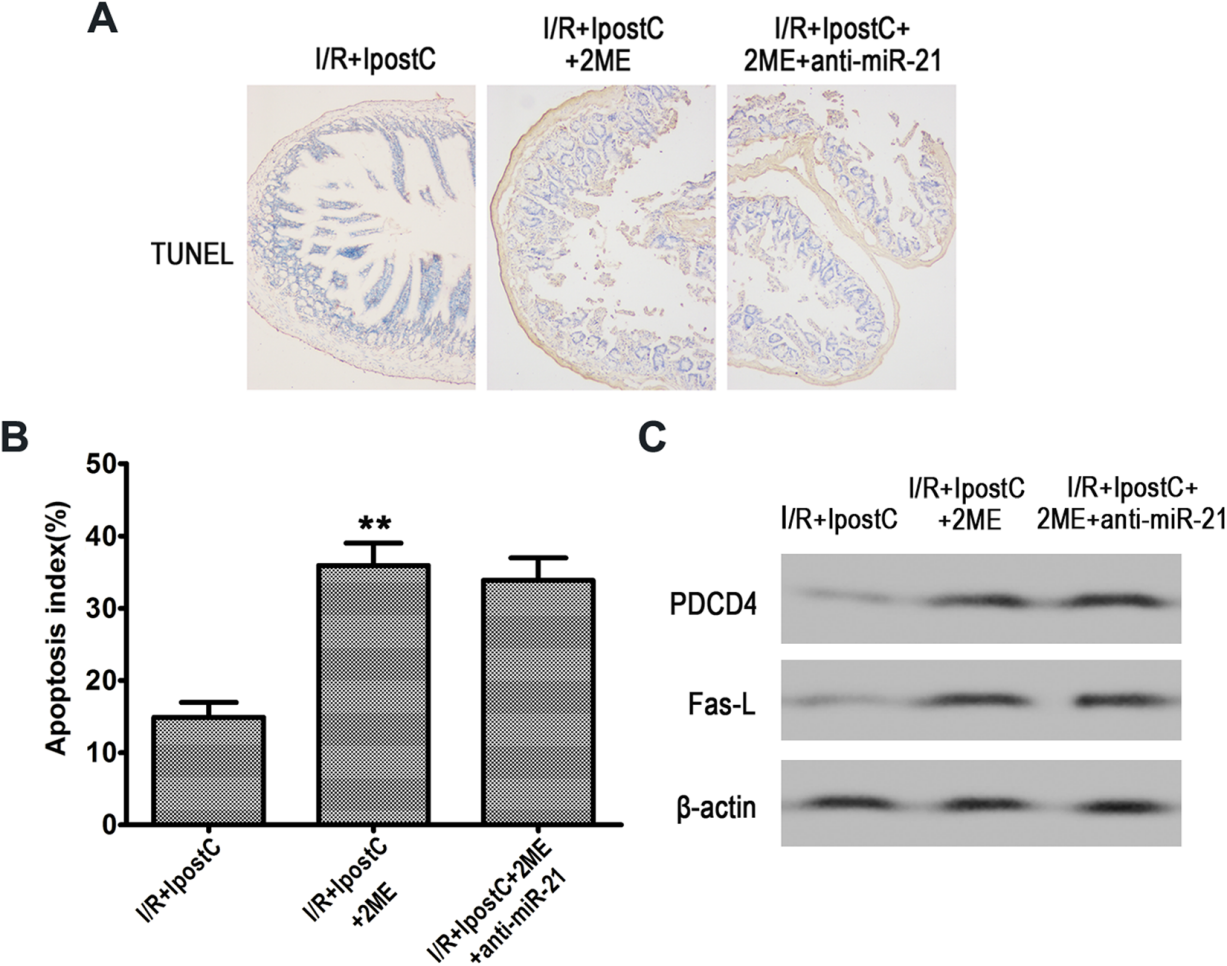
Inhibition of HIF-1α abolished the IPostC-induced suppression of apoptosis. (**A** and **B**) TUNEL staining and apoptotic index in the different groups. Magnification ×200. (**C**) Western blot analysis of PDCD4 and Fas-L expression using β-actin as the internal control. Unprocessed original scans and of blots are shown in Supplementary Fig. S1. Data are expressed as mean ± SD. n = 6. ^**^ *P* < 0.01 compared with I/R + IPostC group.

The involvement of HIF-1α was assessed by treating rats exposed to IPostC with the HIF-1α inhibitor 2-ME, which significantly decreased HIF-1α levels in the nucleus compared with the vehicle group (Fig. 5A; *P* < 0.01). Knockdown of miR-21 had no effect on HIF-1α expression. Inhibition of HIF-1α resulted in a significant downregulation of miR-21 (0.39 ± 0.05 vs 1.0 ± 0.11; *P* < 0.01) concomitant with a significant increase in the intestinal injury score (3.3 ± 0.32 vs 1.3 ± 0.19; *P* < 0.01) in rats exposed to IPostC, and this effect was not enhanced by treatment with anti-miR-21 (Fig. 5B–D), indicating that the protective effects of IPostC may be mediated by a regulatory loop involving HIF-1α and miR-21 in response to hypoxia. To further examine the role of apoptosis in I/R-induced intestinal injury and the protective effects of IPostC, we examined TUNEL-stained sections and assessed the expression of PDCD4 and Fas-L. HIF-1α inhibition increased the apoptosis index (35.9 ± 3.1% vs 14.9 ± 2.1%; *P* < 0.01) and upregulated PDCD4 and Fas-L in tissues exposed to IPostC, whereas knockdown of miR-21 under these conditions had no effect on apoptosis or PDCD4 and Fas-L expression (Fig. 6A–C). Taken together, these data indicated that IPostC inhibits apoptosis via the HIF-1α-mediated modulation of miR-21 and its downstream targets.

## Discussion

The protective effects of IPostC were originally described in the heart of an *in vivo* dog model, where it was shown to reduce neutrophil accumulation and improve endothelial function^17^. Subsequent studies suggested possible mechanisms of IPostC-mediated cardioprotection, such as the attenuation of reactive oxygen species (ROS) production, activation of prosurvival kinase pathways such as ERK1/2 and phosphoinositol 3-kinase/Akt, and mitochondrial modulation^18–20^. In the intestine, I/R injury can lead to tissue damage associated with the generation of oxygen free radicals, neutrophil infiltration, and mucosal apoptosis^21^. However, the mechanisms underlying the protective effects of IPostC against intestinal I/R remain unclear. Here, we used a rat model of intestinal I/R to study the involvement of miR-21 in IPostC and explored the underlying mechanisms.

A recent prospective clinical study of patients undergoing valve replacement with or without IPostC analyzed apoptosis-related miRNAs and identified miR-21 as a differentially regulated miRNA, suggesting its involvement in the mechanism of IPostC^12^. In a rat model of myocardial infarction, miR-21 was shown to be downregulated in infarcted areas, and its overexpression inhibited apoptosis through the modulation of PDCD4^22^. miR-21 is downregulated in cardiomyocytes in response to hypoxia, and its overexpression decreases infarct size, exerting an antiapoptotic effect by suppressing the expression of Fas-L^16^. The protective effect of ischemic preconditioning in the heart was shown to be mediated by the upregulation of miR-21 and the downregulation of its target PDCD4^10^. However, studies have also shown that overexpression of miR-21 for longtime leads to fibrosis in the kidney^23^.

In the present study, we showed that IPostC upregulated miR-21 concomitant with a reduction in I/R injury in a rat model of intestinal I/R. Furthermore, the upregulation of miR-21 by IPostC was associated with the activation of HIF-1α, the suppression of apoptosis, and the downregulation of PDCD4 and Fas-L.

We examined the levels of HIF-1α during I/R and IPostC to clarify the roles of hypoxia, HIF-1α, miR-21, and apoptosis in IPostC. Our results showed that HIF-1α was upregulated in intestinal tissues exposed to I/R, whereas IPostC reduced the injury score and apoptosis concomitant with a further upregulation of HIF-1α and miR-21. The upregulation of HIF-1α and miR-21 by hypoxia was abolished by siRNA-mediated silencing of HIF-1α, confirming the modulation of miR-21 expression by HIF-1α. This modulation of miR-21 by hypoxia-induced HIF-1α was shown previously in human pancreatic cancer cells, where the regulation of miR-21 by HIF-1α decreased apoptosis and promoted cell survival^24^, similar to the findings of the present study.

The cardioprotective effects of the HIF-1α/miR-21 axis were reported by Liu *et al*.^25^, who showed that hypoxia induced miR-21 and HIF-1α, and knockdown of HIF-1α or miR-21 induced cardiomyocyte apoptosis. Similar to the hypoxic tumor microenvironment, myocardial ischemia is characterized by hypoxia, and the consequent activation of HIF-1α plays a critical role in triggering cellular mechanisms of protection against the consequences of oxygen deprivation in the myocardium. HIF-1α is a heterodimeric transcription factor, the levels of which are regulated by cellular oxygen concentration, and its role in the maintenance of oxygen homeostasis has been demonstrated previously^13,26^. The results of the present study showing the upregulation of HIF-1α during IPostC suggest its involvement in the mechanism of IPostC. Zhao *et al*.^27^ showed that IPostC upregulated HIF-1α and hypothesized that repetitive periods of reperfusion and ischemia may result in temporary hypoxia and thus the induction of HIF-1α. Our results were consistent with these findings and suggest a potential mechanism underlying the protective effects of IPostC mediated by the induction of miR-21by HIF-1α, leading to the downregulation of PDCD4 and the inhibition of apoptosis. Future studies should be aimed at exploring the involvement of survival pathways such as PI3K/Akt and ERK1/2 in the effects of IPostC mediated by miR-21 in the intestine, as suggested in previous studies of the heart.

Apoptosis is an important mechanism of cell death in the intestine induced by I/R^28^. IPostC has previously been shown to attenuate I/R-induced intestinal injury by inhibiting apoptosis in the intestinal mucosa in a rat model^21^. These effects of IPostC on alleviating intestinal injury and mucosal cell apoptosis have been shown to be mediated by the Janus kinase/signal transducer and activator of transcription (JAK/STAT) pathway, which plays an important role in I/R-induced intestinal injury^29^. In the present study, we showed that IPostC suppressed apoptosis and I/R injury by upregulating miR-21 and downregulating PDCD4 and Fas-L. miR-21 was shown to protect rat hearts against I/R injury by decreasing apoptosis via the modulation of PDCD4^10^. However, Gao *et al*.^12^ failed to show the involvement of PDCD4 in IPostC-induced cardioprotection, indicating that IPostC attenuated apoptosis by regulating miR-21 and the downstream effectors BCL2 and BAX without affecting PDCD4 levels. Additional studies specific to I/R injury in the intestine are necessary to clarify the mechanisms underlying the effects of IPostC.

In normoxic condition, HIF-1α was hydroxylated by prolyl hydroxylase domain (PHD) proteins, and subsequently degraded via the recruitment of an ubiquitin-protein ligase von Hippel-Lindau (VHL)^30^. Whereas in hypoxic condition, the hydroxylation of HIF-1α by PHD was decreased, resulting in the HIF-1α activation^31^. As a transcription factor, HIF-1α could activate the expression of numerous miRNAs under hypoxia^32^, via the modulation of their transcription factors^33^, include miR-21. Liu, *et al*.^25^ showed that HIF-1α regulate the expression of miR-21 in response to hypoxia, via the binding sites 1 on miR-21 promoter in cardiomyocytes.

miR-21 and HIF-1α was found to be an anti-apoptotic miRNA and factor. Upregulation of HIF-1α and miR-21 in hypoxic condition should be a protective mechanism against hypoxia, however it could not resist the damage caused by hypoxia. Intermittent hypoxia could further up-regulate the expression of HIF-1α and miR-21, making it more likely to counteract the damage caused by hypoxia. But this short time up-regulation is different from that caused by the overexpression vector transfection.

## Conclusion

IPostC attenuates intestinal I/R injury by suppressing apoptosis via a regulatory axis involving the induction of miR-21 by HIF-1α, leading to the downregulation of the apoptotic mediators PDCD4 and Fas-L. Further research is necessary to confirm these findings and to clarify the role of other survival mediators and pathways in the protective effect of IPostC against intestinal injury.

## Methods

### Animals and surgical procedure

The study was approved by the Animal Care Committee of TongJi University, and all animal procedures were performed in compliance with National Institutes of Health guidelines. Sixty adult pathogen-free male Sprague-Dawley rats weighing 220 to 250 g were housed in individual cages in a temperature-controlled room with 12-hour light-dark cycles and free access to water ad libitum.

Rats were anesthetized with 20% urethane (6 mL/kg) intraperitoneally, tracheostomized, and mechanically ventilated with room air. A midline laparotomy was performed to access the small intestine. Intestinal ischemia was established by occluding the superior mesenteric artery (SMA) and confirmed by immediately blanching the small intestine and cecum. A return to original color indicated restoration of blood flow to the gut after declamping of the SMA. Normothermia (36 °C–38 °C) was maintained with heating pads during the procedure.

### Experimental protocol

Rats were randomly divided into the following groups: (1) sham operation group, n = 6; (2) I/R group, n = 12; (3) IPostC + I/R group, n = 18; (4) anti-miR-21 + I/R group, n = 6; (5) anti-miR-21 + IPostC + I/R group, n = 6; (6) 2-methoxyestradiol (2ME) + IPostC + I/R group, n = 6; and (7) 2ME + anti-miR-21 + IPostC + I/R group, n = 6. In the sham operation group, the SMA was separated, but without occlusion. In the I/R groups, SMA occlusion time was 60 minutes and reperfusion time was 2 hours. In the IPostC groups, the SMA was occluded by 3 cycles of 30-second reperfusion before another reperfusion as described previously^7^.

### Cell culture and conditions

Intestinal epithelial cells (IEC-6) were obtained from the American Type Culture Collection (ATCC; Manassas, VA, USA) and cultured in Dulbecco’s Modified Eagle’s Medium supplemented with 4.5 g/L D-glucose, 10% v/v fetal bovine serum, and 1% penicillin/streptomycin (Gibco; Invitrogen Ltd., Shanghai, China) at 37 °C in a 5% CO_2_ and 21% O_2_ incubator. Hypoxia was induced by culturing cells in a modular incubator equipped with an O_2_ sensor, which enabled the mixing of N_2_ and air to achieve hypoxic conditions (10% or 2% O_2_ and 5% CO_2_ balanced with N_2_). For reoxygenation, cells were transferred to normoxic conditions.

### Construction of adenoviruses

The adenoviruses expressing PDCD4 (Ad-PDCD4), Fas-L (Ad-Fas-L), or GFP (Ad-GFP; control adenovirus) were generated using the Adeno-XTM Expression Systems 2 kit (Clontech, CA) according to the manufacturer’s protocols^22^. These adenoviruses were purified by cesium chloride gradient ultracentrifugation and were titrated using a standard plaque assay.

### Cell transfection

To knockdown HIF-1α expression, the synthesized siRNA oligonucleotides against HIF-1α were transfected in cells, along with the scrambled oligonucleotides. After transfection for 48 h, the cells were harvested and subjected to western blotting to analyze HIF-1α expression. β-actin was used as an internal control. For miR-21 knockdown, a miR-21 inhibitor (LNA-anti-miR-21; Exiqon, Woburn, MA, USA) was added to the culture medium at a final concentration of 30 nM. For miR-21 upregulation, pre-miR-21 (Ambion, Grand Island, NY, USA) was added directly to the complexes and used at a final concentration of 30 nM. PDCD4 and Fas-L gene upregulation was performed by Ad-PDCD4 (30 MOI) and Ad-Fas-L (30 MOI), respectively. The transfection medium was replaced with regular culture medium 4 h post-transfection. Vehicle control, oligo controls for anti-miR-21 (Exiqon) and pre-miR-21 (Ambion), and adenovirus control (Ad-GFP) were applied.

### *In vivo* miRNA knockdown using LNA-modified anti-miR

Locked nucleic acid (LNA)-modified anti-miR-21 oligonucleotides (Exiqon) were diluted in saline (5 mg/mL) and administered into the tail vein (10 mg/kg) less than 1 hour before ischemia surgery.

### Inhibition of HIF-1α in the rat model

The HIF-1α inhibitor 2-ME (Sigma-Aldrich; St. Louis, MO, USA) was dissolved in DMSO (2%, Sigma-Aldrich) and diluted with arachis oil. Rats received intraperitoneal injections of 2-ME at a final dose of 100 mg/kg at 24 hours before ischemia. The protein expression levels of HIF-1α were measured with western blotting, and the expression of miR-21 was measured with real-time PCR.

### Scoring of intestinal mucosal injury

Sections of the small intestine were stained with hematoxylin-eosin (HE) and examined with light microscopy. Damage to the intestinal mucosa was evaluated in a blinded manner by two independent pathologists. The degree of injury was scored according to the method described by Chiu *et al*.^34^ with this method, the score is based on changes in the villi and glands of the intestinal mucosa. In brief, mucosal damage was graded as follows: grade 0: normal mucosal villi; grade 1: development of subepithelial gruenhagen space at the apex of the villi and capillary congestion; grade 2: extension of the subepithelial space with moderate lifting of the epithelial layer from the lamina propria; grade 3: extensive epithelial lifting with few denuded tips; grade 4: denuded villi with exposure of lamina propria and dilated capillaries and increased cellularity of the lamina propria; and grade 5: digestion and disintegration of the lamina propria, hemorrhage, and ulceration.

### Intestinal mucosal epithelial apoptosis evaluation

Apoptosis was quantified using an *in situ* apoptosis detection kit (Roche, Switzerland) as described previously^27^. Briefly, transmural intestinal tissue blocks were fixed using 4% paraformaldehyde in PBS, embedded in paraffin, and subjected to TUNEL staining according to the manufacturer’s protocol. The apoptosis index was determined by measuring the percentage of apoptotic nuclei from the total nuclei in each slide.

### Western blotting

For western blot detection of the HIF-1α protein, tissue blocks (50 mg) were lysed and homogenized. The protein concentration in the supernatant was determined using the BCA method. Protein samples (20 μg) were separated through 8% sodium dodecyl sulfate-polyacrylamide gel electrophoresis and transferred onto nitrocellulose membranes. Membranes were blocked with 3% bovine serum albumin for 2 hours at room temperature and then incubated with primary antibody at 4 °C overnight. The membranes were incubated with horseradish peroxidase-conjugated goat anti-mouse IgG (1:1,000 polyclonal) at room temperature for 1 hour. The primary antibodies used were anti-mouse monoclonal HIF-1α (1:200, Santa Cruz Biotechnology; Santa Cruz, CA, USA), anti-mouse monoclonal β-actin (1:1,000, Santa Cruz Biotechnology), and anti-mouse monoclonal LAMIN-B (1:100, Santa Cruz Biotechnology). Bands were detected using electrochemiluminescence.

### Taqman real-time PCR

Total RNA was isolated from tissues using the Trizol reagent (Invitrogen; Carlsbad, CA, USA), and miRNA was quantified by real-time PCR with Taqman chemistry (Applied Biosystems; Carlsbad, CA, USA) as described previously^35^. As an internal control, U6 was used for template normalization for miR-21 expression.

### Statistical analysis

The results were expressed as means ± SD and analyzed with SPSS 13.0 software (SPSS Inc; Armonk, NY, USA). One-way ANOVA was used for statistical evaluation of the data. *P* values < 0.05 were considered significant.

## Electronic supplementary material

Supplementary information
