# Supplementary material for: Ischemic Postconditioning Protects Against Intestinal Ischemia/Reperfusion Injury via the HIF-1α/miR-21 Axis
Source: Sci Rep. 2017 Nov 23;7:16190. doi: 10.1038/s41598-017-16366-6 (PMC5700993; doi:10.1038/s41598-017-16366-6)
Supplement: Supplementary file 1 — Supplementary information [file 41598_2017_16366_MOESM1_ESM.pdf]

Ischemic Postconditioning Protects Against Intestinal Ischemia/Reperfusion Injury via the HIF-1α/miR-21 Axis

Zhongzhi Jia[1*], Weishuai Lian[2*], Haifeng Shi[1], Chuanwu Cao[2], Shilong Han[2], Kai Wang [1], Maoquan Li[2, 3] & Xiaoping Zhang[3]

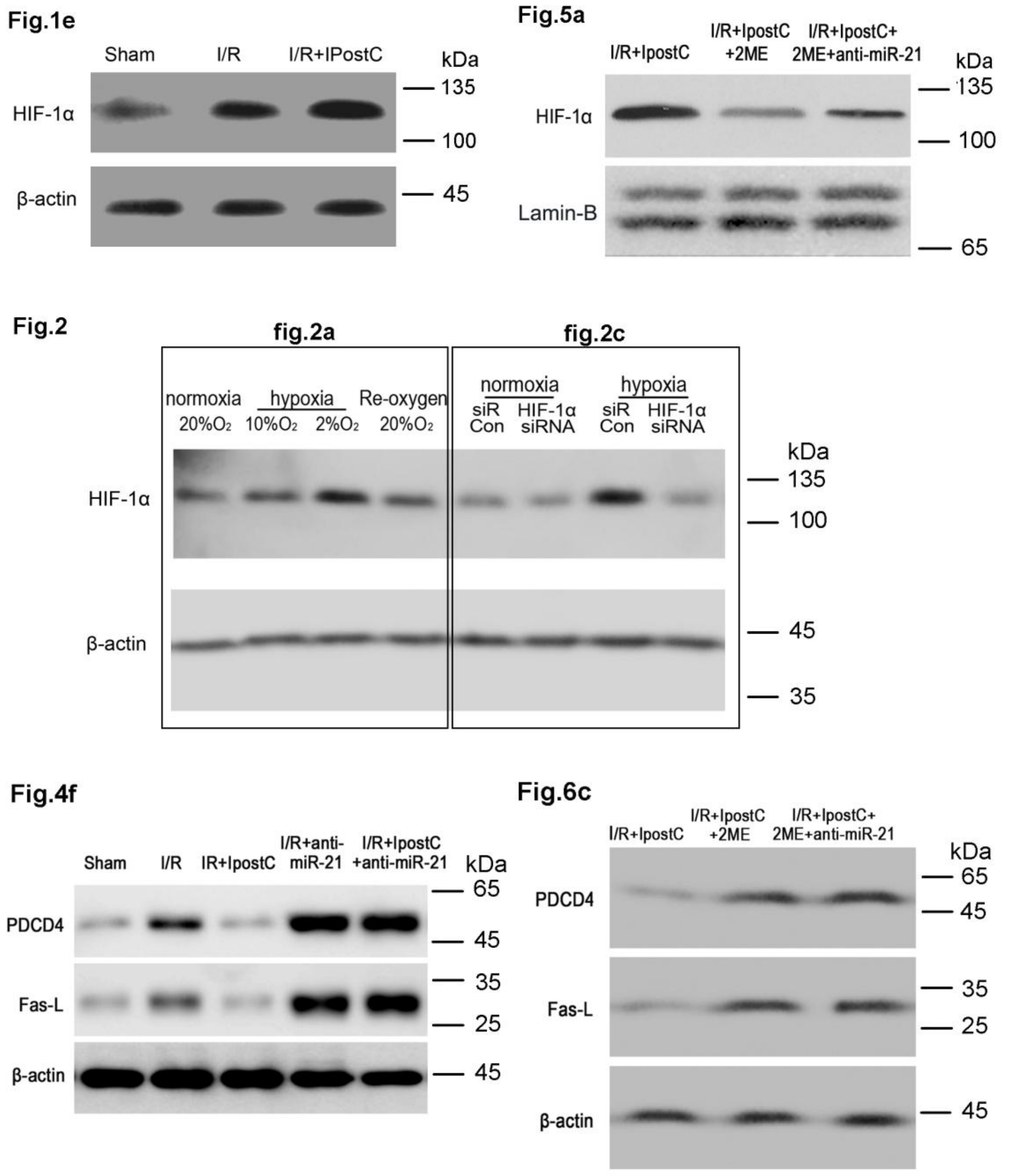

Supplementary Figure. S1. The full blot images, with molecular weight markers indicated, for Figures 1e, 2a, 2c, 4f, 5a and 6c.
